# Fabrication and Characterization of Au-Decorated MCM-41 Mesoporous Spheres Using Laser-Ablation Technique

**DOI:** 10.3390/ma15217470

**Published:** 2022-10-25

**Authors:** Hassan S. Al Qahtani, Sultan Akhtar, Mir Waqas Alam, Mohammad Kamal Hossain, Abbad Al Baroot, Muidh Alheshibri

**Affiliations:** 1 EXPEC Advanced Research Centre, Saudi Aramco, Dhahran 31311, Saudi Arabia; 2 Department of Biophysics, Institute for Research and Medical Consultations (IRMC), Imam Abdulrahman Bin Faisal University, Dammam 31441, Saudi Arabia; 3 Department of Physics, College of Science, King Faisal University, Al-Ahsa 31982, Saudi Arabia; 4 Interdisciplinary Research Center for Renewable Energy and Power Systems (IRC-REPS), Research Institute 5040, King Fahd University of Petroleum and Minerals (KFUPM), Dhahran 31261, Saudi Arabia; 5 Department of Basic Engineering Sciences, College of Engineering, Imam Abdulrahman Bin Faisal University, Dammam 31441, Saudi Arabia; 6 Department of Basic Sciences, Deanship of Preparatory Year and Supporting Studies, Imam Abdulrahman Bin Faisal University, Dammam 31441, Saudi Arabia; 7 Basic & Applied Scientific Research Centre, Imam Abdulrahman Bin Faisal University, Dammam 31441, Saudi Arabia

**Keywords:** laser ablation, liquid media, mesoporous silica spheres, gold target and gold nanoparticles

## Abstract

This study reports the synthesis of Au-decorated MCM-41 mesoporous nanoparticles using a laser-ablation technique. It was observed that the number of Au attached to MCM-41 nanostructures was dependent on the amount of encapsulated Cationic surfactant (cetyl ammonium bromide (CTAB) volume. The chemical group of the prepared nanoparticles was analyzed by FT-IR spectroscopy, where different absorption peaks corresponding to Au and MCM-41 were observed. The observed band region was ∼1090, 966, 801, 2918, and 1847 cm^−1^ for different samples, clearly confirming the successful preparation of MCM-41 with CTAB and Au-decorated MCM-41 nanoparticles using environmentally friendly laser-ablation approach. The surface morphology of the prepared nanoparticles were performed using TEM techniques. The TEM analysis of the MCM-41 specimen showed silica spheres with an average size of around 200 nm. Furthermore, Raman spectroscopy was done to evaluate the chemical structure of the prepared nanoparticles. It was seen that the prepared Au NPs decorated the MCM-41 system facilitated strong Raman peaks of CTAB. In addition, eight distinct Raman peaks were observed in the presence of Au NPs. This new functionalized method using the laser-ablation approach for mesoporous nanoparticles will participate effectively in multiple applications, especially the encapsulated molecule sensing and detection.

## 1. Introduction

Mesoporous silica nanoparticles (MSN) have attracted both the research and industry communities due to their unique properties, including large surface areas, high loading capacities, thermal stability, and easy surface functionalization. Specifically, the porous nature of nanoparticles allows for the encapsulation and release of materials, such as drugs and surfactants, making MSN ideal candidates for drug/surfactant delivery carriers in multiple applications. MSN have a diameter in the range of 2–50 nm templated from liquid crystalline surfactant micelles [1,2,3]. Apart from drug delivery and protein delivery [4,5], they have recently been used for catalysis [6] in the oil and gas industries as well [7]. In 1992, Mobil crystalline materials (MCM-41) were synthesized by the Mobil Corporation Company to overcome the disadvantages of conventional zeolites and molecular sieves with pore diameters less than 2 nm for more facile conversion of heavy petroleum oil [8,9]. CTAB was used as a template to create pores in a silica framework. Thereafter, various types of surfactants—namely, cationic, anionic, and neutral—have been used to synthesize silica MSN [10]. These surfactants play an important role in defining the pore size and volume of silica MSNs.

Functional groups are always required to prepare stabilized silica-supported metal nanoparticles (NPs). Thus, MCM-41 has been investigated for the preparation of silica-supported noble palladium, iridium, and rhodium nanocatalysts using the surfactant- stabilization approach [11]. MCM-41 containing these noble metals was found to be active and selective of catalysts in the hydrogenation of cyclic olefins, such as cyclohexene, cyclooctene, cyclododecene, and norbornene. Doping MCM-41 with selected metals also showed significant enhanced performance of catalysts by tunning ptCo/MCM-41 metal–metal interactions [12]. The synergy between Pt and Co significantly improved the catalytic performance for the selective oxidation of glycerol to glyceric acid in a base-free medium. This bimetallic doping to MCM-41 increased the catalytic stability in base-free medium with very good catalytic activity. In addition, Yen et al. have synthesized Au–Ag bimetallic NPs supported on mesoporous silica (MCM-41) using the surface-functionalized silica as the supporting host to confine the bimetallic nanoparticles [12]. The bimetallic Ag–Au–MCM-41 catalyst exhibited higher activity for the low-temperature oxidation of CO with high stability compared to the monometallic Ag–MCM-41 and Au–MCM-41 catalysts. Further, Wang et al. synthesized Au NPs coated, PFH-encapsulated and PEGylated mesoporous silica nano-capsule-based nano-platforms for ultrasound contrast imaging, which showed an enhancement in their ultrasound sensitivity [13] to functional gold nanoparticles. Similarly, Wang et al. developed a Janus-system gold-mesoporous act as targeted CT-imaging agents for HCC diagnosis [14]. The synthesized nanoparticles were able to identify the tumor site of the tumor-bearing nude mice.

Functionalizing NPs using pulsed laser ablation in liquids (PLAL) has the great advantage of being environmentally friendly compared to conventional chemical synthesis methods, which use harmful chemical reagents, and avoid surface contamination in surface-related applications, such as surface-enhanced Raman scattering (SERS) [15]. SERS helps overcome the low signal-to-ratio problems of Raman spectroscopy and upgrades the Raman signal. To reach the higher enhanced factors, NPs need to be adjusted to a certain surface plasmon resonance [16]. Thus, nobel metals, such as Au, Ag, Pt, and Cu, are used because they exhibit localized surface plasmon resonance (LSPR) in the visible region caused by the excitation of the conduction electrons after light irradiation [17,18,19]. Researchers have investigated the SERS activities of metal/core-shell NPs [16,20,21,22,23]. The results have shown that metal/core-shell NPs have unique properties as good candidates for SERS applications compared to pure metal or core-shell NPs alone.

In the present work, MCM-41 mesoporous nanoparticles with different rations of encapsulated CTAB surfactants were fabricated and decorated thereafter by Au metal NPs using an environmentally friendly PLAL technique. The population of Au/MCM-41 nanostructures obtained was found to be altered by modifying the encapsulated CTAB volume. The PLAL products prepared under different CTAB volumes were characterized by Fourier transform infrared (FTIR) spectroscopy, transmission electron microscopy (TEM, SAED), thermal gravimetric analysis (TGA), ultraviolet-visible (UV-Vis) spectroscopy, and surface-enhanced Raman scattering (SERS). The results are shown and discussed below in separate sections.

## 2. Materials and Methods

### 2.1. Materials

MCM-41 was synthesized according to a procedure described elsewhere [24]. Briefly, the CTAB (purchased from the Sigma–Aldrich Company, Saint Louis, MO, USA) was dissolved in 480 mL of 18.2 DI water. The mixture was stirred until all the CTAB was dissolved. An NaOH solution (2 M) was prepared in 18.2 DI water, and 3.5 mL was added to the beaker containing CTAB and water; the temperature was then adjusted to 80 °C. After that, 5 mL tetraethylorthosilicate was added dropwise while maintaining vigorous stirring of the solution. The mixture was stirred for two hours. The resultant white precipitate was collected, washed several times with deionized water, and centrifuged at 9000 rev/min for 15 min. The powder was then dried at 50 °C for two days. The CTAB surfactant molecules were extracted by using alcoholic solutions of ammonium nitrate. Typically, 1 g of MCM-41 was dispersed in 150 mL of ethanol (95%) containing 0.3 g of NH_4_NO_3_, and the mixture was stirred at 333 K for 15 min. The amount of NH_4_NO_3_ corresponded to an NH_4_+/CTMA+ molar ratio of 2. Solids were recovered by filtration and washed with cold ethanol. This method repeated twice for removal of all CTAB encapsulated within MCM-41 while in this study have been done once only for extracting half the CTAB amount encapsulated inside MCM-41 [25].

High pure (99.99%) gold was used as the main target in this study. High-grade purified water (ELGA PURELAB Chorus 3 system with a resistivity of 18.2 MΩ) was used. The target and all glassware were used after being carefully cleaned with acetone, followed by extensive rinsing with distilled water.

### 2.2. Preparation Methods

#### Au Factualization of MCM-41 Mesoporous Nanoparticles via PLAL Technique

**Figure 1:**
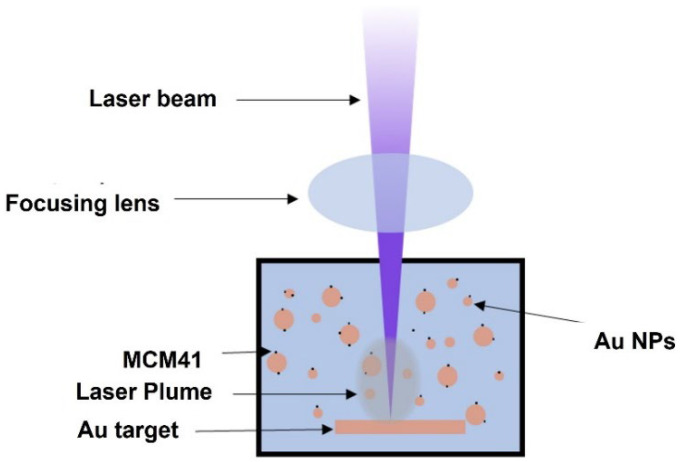
Schematic illustration of the use of the PLAL method to prepare Au-decorated MCM-41 NPs.

Laser ablation was carried out using a Q-switched Nd:YAG pulsed laser (PS-2225, LOTIS TII Ltd.; Minsk, Belarus) with a wavelength of 1064 nm. The target was placed at the bottom of a 20 mL glass container filled with 10 mL of a solution of different ratios of CTAB surfactant incapsulated inside the MCM-41 mesoporous nanoparticle suspension to fabricate Au–MCM-41–CTAB, and Au–MCM-41–50% CTAB nanoparticles. The vertical distance from the target was 10 mm. The process of ablation was achieved with a 10 Hz repetition rate, 5.4 J/cm^2^ laser fluence, and a pulse width of 10 ns for 15 min. The distance between the target and the lens was set to be lower than the focal length to obtain a larger spot size of 1.5 mm on the target. The laser was focused on the target by plano-convex lens of 100 mm. During laser ablation, a magnetic stirrer, which rotates at 300 rpm, was placed at the bottom of the container to maintain a steady interaction between the laser and the metal target. The scheme for the PLAL set-up technique is shown in Figure 1.

Transmission electron microscopy (TEM) is a high-resolution tool used to study the morphology and structure of nanomaterials. To reveal the attachment or coupling of Au NPs onto silica spheres, the PLAL-prepared materials were characterized using TEM (Morgagni 268, FEI at 80 keV) methods. For this purpose, the dispersion of prepared products (pure MCM-41, Au–MCM-41-encapsulated CTAB, and Au–MCM-41-encapsulated 50% CTAB) were deposited onto TEM grids coated with lacy carbon film (with nano-sized holes) [26]. The TEM grids were dried before being transferred onto the microscope for examination. The particle size of both the gold nanoparticles (Au NPs) and MCM-41 nanoparticles were measured from TEM images using Gatan digital micrograph software and represented as size histograms. Furthermore, the crystalline nature of the prepared nanoparticles/silica spheres was studied by using electron diffraction pattern as selected area electron diffraction (SAED). The optical properties of the Au-NP-decorated MCM-41 and pristine MCM-41 were obtained under different CTAB concentrations, and conditions were investigated using the JASCO UV-VIS-NIR Spectrophotometer (V-670) measurement system. Each sample was placed in a quartz cuvette with a standard optical path length of 10 mm. The absorbance spectra were recorded between 200 and 800 nm. The bonding of the MCM41 samples was studied using Fourier transform infrared spectroscopy (FTIR) (Nicolet 6700, FTIR spectrometer). The FTIR spectra were recorded between 4000 and 400 cm^−1^. Raman spectra were obtained using the LabRAM HR Evolution Raman system (30–4000 cm^−1^) equipped with an internal HeNe 17 mW laser at a 633-nm excitation wavelength. The laser was filtered to 25% to avoid dissociation and damage to the sample. A long working distance lens (50×) was used to focus the excitation on the sample, and scattered photons were collected in a backscattering configuration for 10 s and accumulation of 2 at a grating of 600 g/mm. The microscope was coupled confocally to an 800-mm focal length spectrograph equipped with two switchable gratings (1800 g/mm and 600 g/mm). Thermal gravimetric analysis (TGA) was performed to quantify the organic content present using the Q500 TA Instruments thermal analyzer with a heating rate of 10 °C min^−1^ from 25 °C to 900 °C under nitrogen flow (10 mL min^−1^). The instrument has an isothermal accuracy of ±1 °C. The sample was crushed evenly, and about 10 mg of the powder was placed on the platinum pan and loaded into the TGA.

## 3. Results and Discussion

### 3.1. TGA Analysis

**Figure 2:**
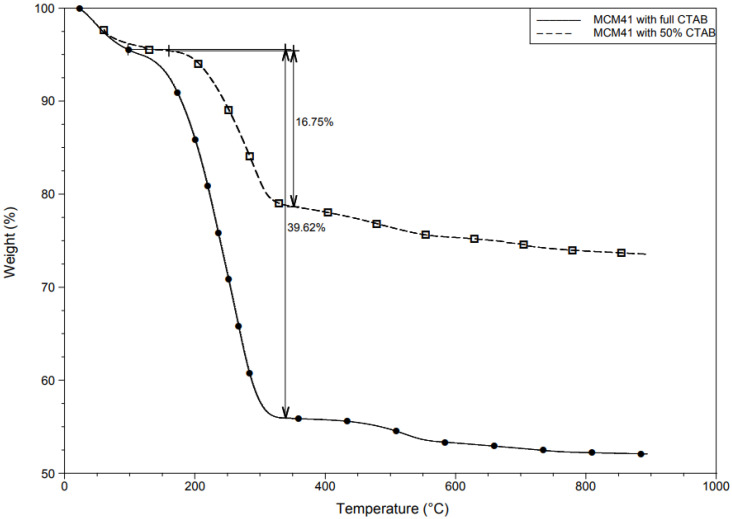
TGA results for the encapsulated surfactant in MCM-41 indicate 40% of total organic content in MCM-41 with the full CTAB sample, while approximately 20% of MCM-41 with the 50% CTAB sample.

MCM-41 is composed mainly of silicon dioxide and surfactant, where the decomposition of the material can be used to indicate the amount of organic surfactant encapsulated. As shown in Figure 2, the weight loss from 30 °C to 150 °C was a result of the desorption of the physically adsorbed water [25]. Moreover, CTAB has been reported as beginning to decompose at around 200 °C [27]. Hence, the weight loss from 200 °C to 300 °C indicates the initial amount of surfactant in the nanocapsules. As shown in the figure, approximately 40% of the total mass of the material was organic content in MCM-41 with a full CTAB (100% CTAB)-encapsulated sample and 20% in MCM-41 with a 50% CTAB sample. The decomposition of silicon dioxide was greater than 1000 °C.

### 3.2. TEM Analyses

**Figure 3:**
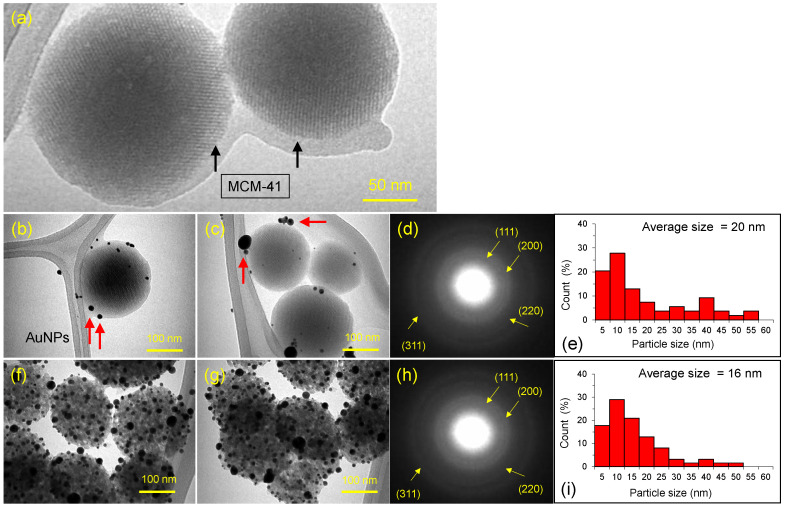
TEM images, SAED patterns, and size histogram of PLAL-prepared Au–MCM-41–CTAB composite; (**a**) pure MCM-41, (**b**–**e**) Au–MCM-41–full CTAB, and (**f**–**i**) Au–MCM-41–50% CTAB.

TEM was performed to reveal the AuNPs attachment on the surface of the MCM-41. Figure 3 shows the TEM analysis of the pure MCM-41, Au–MCM-41–CTAB, and Au–MCM-41–50% CTAB specimens. The TEM of the pure CTAB specimen disclosed the obvious structure and shape of the silica spheres (Figure 3a), two such spheres are marked with black arrows. The average size of the spheres was estimated around 200 nm. Upon adding the AuNPs during the PLAL experiment, the prepared product (Au–MCM-41 specimen) showed the Au NP attachment with the MCM-41 spheres; in contrast, the Au NPs were observed as attached or well-connected to the silica spheres (Figure 3b,c). The Au NPs appeared darker than the silica MCM-41 spheres due to their higher atomic number compared to effective mass of Si and O (SiO_2_). The Au NPs attached to silica spheres are marked with red arrows. According to the Lifshitz-Slyozov-Wagner (LSW) model, nanoparticles (NPs) follow a reaction-limited growth mechanism at low CTAB concentrations and a diffusion-limited growth mechanism at high CTAB concentrations. As expected, the number of attached Au NPs was higher when the concentration of CTAB was reduced to 50% (Au–MCM-41–50% CTAB specimen), and the ratio of Au NPs was increased (Figure 3f,g). In this case, the silica MCM-41 spheres were fully covered by Au NPs, and their size was more uniform than the sample encapsulated with Au–MCM-41–full CTAB. The average size of the Au NPs was found to be 20 (Au–MCM-41–CTAB; Figure 3e) and 16 nm (Au–MCM-41–50% CTAB; Figure 3i). The crystalline structure of the particles was verified by analyzing the diffraction patterns (SAED patterns) of both samples (Figure 3d,h). The first four diffraction reflections (d-spacings) in the form of rings are highlighted with yellow arrows and were indexed as (111), (200), (220), and (311)—suggesting the face-cantered cubic (fcc) structure of the Au NPs (JCPDS: 04–0784) [28,29].

### 3.3. UV-Vis and FTIR Spectroscopy Analysis

**Figure 4:**
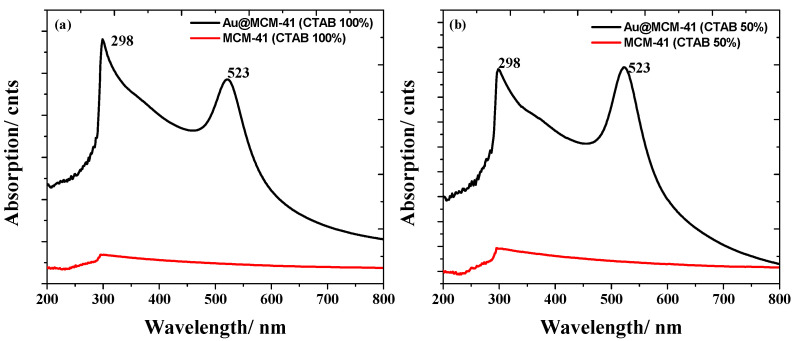
(**a**) UV-Vis absorption of Au–MCM-41–full CTAB and pure MCM-41–full CTAB (100% CTAB); (**b**) the same for Au–MCM-41–50% CTAB and pure MCM-41–50% CTAB.

Distinct absorption peaks for Au and MCM-41 were observed in the UV-Vis absorption measurements, as shown in Figure 4. Figure 4a represents the absorption spectra of Au–MCM–full CTAB and pure MCM-41–full CTAB. The peak at 523 nm corresponded to that of the Au NPs, whereas the peak at 298 nm confirmed MCM-41 [30]. Figure 4b displays the absorption spectra of Au–MCM-41–50% CTAB and pure MCM-41–50% CTAB; the peaks for the Au NPs and MCM-41 were observed to be at 523 nm and 298 nm, respectively.

**Figure 5:**
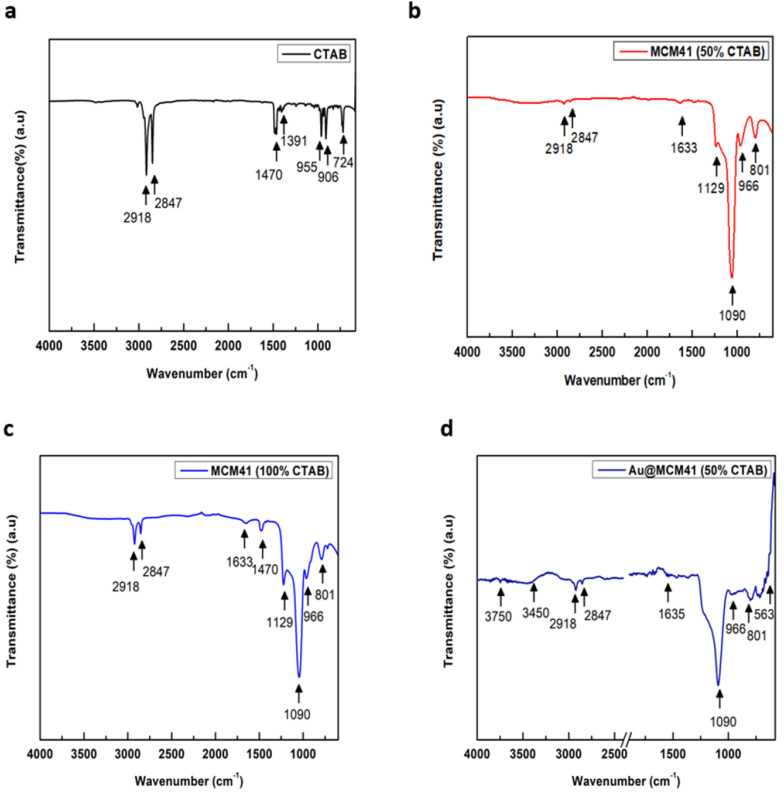

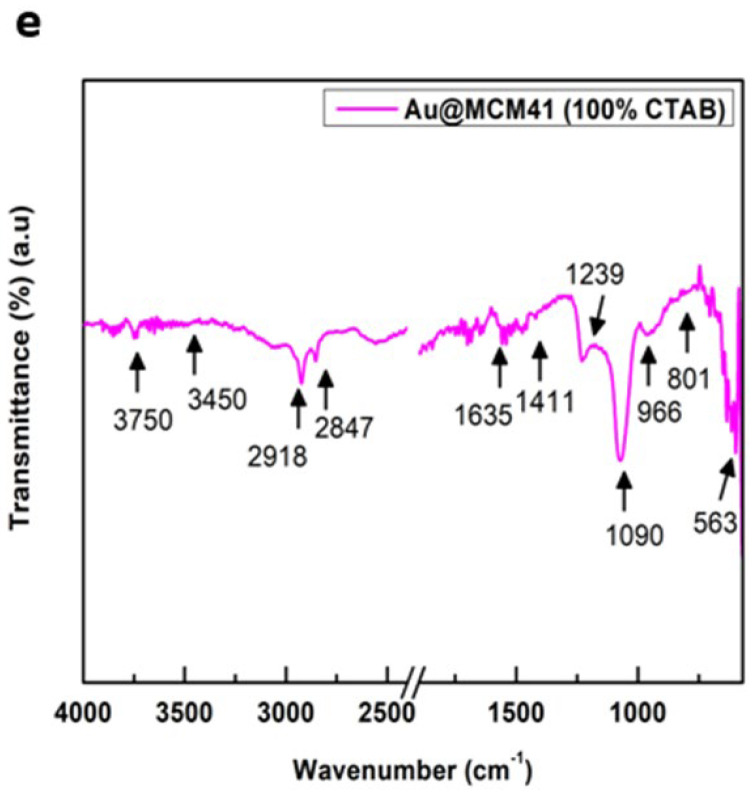
FTIR spectra of (**a**) CTAB surfactant only; (**b**) MCM-41 (50% CTAB); (**c**) MCM-41 (100% CTAB); (**d**) Au–MCM-41 (50% CTAB); and (**e**) Au–MCM-41 (100% CTAB).

The FTIR spectra of MCM-41 and the pulsed laser ablation of the Au NP–decorated MCM-41 with different percentages of CTAB are shown in Figure 5. As shown, the range of data was drawn between 4000 and 400 cm^−1^. The FTIR spectrum shows two strong peaks in Figure 5a at 2918 and 2847 cm^−1^, which are assigned to secondary amine group stretching, C–H anti-stretching, and C–H stretching, respectively, as demonstrated by Nithiyanantham et al. [31]. Additionally, four minor peaks at 1470, 955, 906, and 724 cm^−1^ are due to CTAB powder only, as shown in Figure 5a [31]. However, we did not observe strong peaks in the range of 3450 to 3750, as shown in Figure 5a–c, which are assigned to the absent molecule of water but we have observed minor peaks in these ranges in Figure 5d,e due to the present molecule of water. In Figure 5b,c, one strong peak is observed at 1090 cm^−1^ due to the presence of MCM-41, as investigated by Siddiqui et al. [32], which are assigned to the stretching vibrations of Si–O–Si; however, the two minor peaks at 966 and 801 cm^−1^ indicated symmetric and anti-symmetric stretching vibrations of Si–O bonds cm^−1^ as reported by Siddiqui et al. [32]. However, as we see from Figure 5b,c, the intensity of the peaks in the range 2918 and 2847 increased gradually due to a higher percentage of CTAB in MCM-41. Figure 5d,e show the FTIR spectra of the pulsed laser ablation of Au NP–decorated MCM-41 with different percentages of CTAB. The broad bands at 1635 cm^−1^ indicate to the carbonyl groups (C=O), whereas the bands observed in the range of 1411 and 1239 cm^−1^ indicated stretching vibrations of aliphatic C–H groups in extracts, as reported by F. Mahmoudi et al. [33]. The small changes in intensity and wave number of MCM-41 related peaks mentioned earlier were due to interactions between Au NP–decorated MCM-41 with CTAB. We noted that the four samples in Figure 5b–e showed the characteristic band at the region (∼1090; 966 and 801 cm^−1^; 2918 and 2847), confirming the successful preparation of MCM-41 with CTAB and Au-decorated MCM-41 nanoparticles using the laser-ablation approach in the band (563, 1411, and 1635 cm^−1^) indicated peaks related to Au as well, as demonstrated by F. Mahmoudi et al. [33].

### 3.4. Raman Spectroscopy Analysis

**Figure 6:**
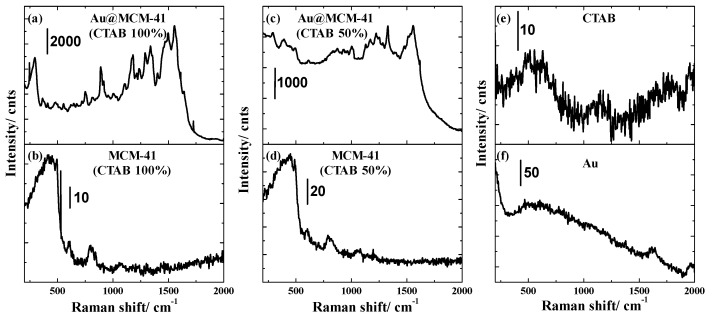
(**a**–**f**) Raman spectra of Au–MCM-41 (CTAB 100%), MCM-41 (CTAB 100%), Au–MCM-41 (CTAB 50%), MCM-41 (CTAB 50%), CTAB and Au, respectively.

We observed that the Au NP–decorated MCM-41 system facilitated strong Raman peaks of CTAB, as shown in Figure 6. Figure 6a,b represent the Raman spectra of Au-decorated MCM-41 and that of MCM-41 only. Eight distinct Raman peaks were observed in the presence of Au NPs, as shown in Figure 6a. These peaks of CTAB demonstrated clearly after the Au has been attached into MCM-41 encapsulated CTAB. The Raman bands are tabulated in Table 1, along with band assignments. Most of the bands coincided with the reported values [34,35,36]. The core CTAB was reduced to 50% of the initial amount and the as-fabricated MCM-41 was decorated with Au NPs. Figure 6c,d display the Raman spectra of Au-decorated MCM-41 (CTAB 50%) and that of MCM-41 (CTAB 50%) only. For reference, the Raman spectra of CTAB and aqueous Au NPs only were extracted and are shown in Figure 6e,f.

## 4. Conclusions

In this study, Au-decorated MCM-41 mesoporous nanoparticles were synthesized using a laser-ablation technique. The amount Au NPs attached to MCM-41 nanostructures was found to be affected by the encapsulated CTAB volume. Structural, optical and morphological characterization was performed by Fourier transform infrared (FTIR) spectroscopy, transmission electron microscopy (TEM, SAED), thermal gravimetric analysis (TGA), ultraviolet-visible (UV-Vis) spectroscopy, and surface-enhanced Raman scattering (SERS). The Raman results showed the effectiveness of the new laser-ablation functionalization approach to the detection of the encapsulated molecules.

## Figures and Tables

**Table 1:** Raman bands of CTAB observed under this investigation and corresponding band assignments.

Raman Band of CTAB (cm^−1^)	Tentative Band Assignments
1555	CH_3_ deformation
1496	CH_2_ scissors
1338	CH_2_ wag
1286	CH_2_ twist
1176	C-C deformation
891	CH_3_ deformation
753	CN^+^ stretch
294	C_4_N^+^ deformation

## Data Availability

All datasets used for this study are included in the manuscript.
